# Cross-sectional interview study of fertility, pregnancy, and urogenital schistosomiasis in coastal Kenya: Documented treatment in childhood is associated with reduced odds of subfertility among adult women

**DOI:** 10.1371/journal.pntd.0006101

**Published:** 2017-11-27

**Authors:** Sarah C. Miller-Fellows, Laura Howard, Rebekah Kramer, Vanessa Hildebrand, Jennifer Furin, Francis M. Mutuku, Dunstan Mukoko, Julianne A. Ivy, Charles H. King

**Affiliations:** 1 Department of Anthropology, Case Western Reserve University, Cleveland, Ohio, United States of America; 2 Department of Environment and Health Sciences, Technical University of Mombasa, Mombasa, Kenya; 3 Vector-borne Disease Unit, Ministry of Health, Nairobi, Kenya; 4 Center for Global Health and Diseases, Case Western Reserve University, Cleveland, Ohio, United States of America; 5 Department of Medicine, Case Western Reserve University, Cleveland, Ohio, United States of America; Swiss Tropical and Public Health Institute, SWITZERLAND

## Abstract

**Background:**

Previous research has documented an increased risk of subfertility in areas of sub-Saharan Africa, as well as an ecological association between urogenital schistosomiasis prevalence and decreased fertility. This pilot project examined reproductive patterns and the potential effects of childhood urogenital *Schistosoma haematobium* infection and individual treatment experience on adult subfertility among women who were long-term residents in an *S*. *haematobium*-endemic region of coastal Kenya.

**Methodology/Principal findings:**

We analyzed findings from 162 in-depth interviews with women of childbearing age in a rural, coastal community, linking them, if possible, to their individual treatment records from previous multi-year longitudinal studies of parasitic infections. Reproductive histories indicated a much local higher local rate of subfertility (44%) than worldwide averages (8–12%). Although, due to the very high regional prevalence of schistosomiasis, a clear relationship could not be demonstrated between a history of *S*. *haematobium* infection and adult subfertility, among a convenience sub-sample of 61 women who had received documented treatment during previous interventional trials, a significant association was found between age at first anti-schistosomal treatment and later fertility in adulthood, with those women treated before age 21 significantly less likely to have subfertility (*P* = 0.001).

**Conclusions/Significance:**

The high subfertility rate documented in this pilot study suggests the importance of programs to prevent and treat pelvic infections in their early stages to preclude reproductive tract damage. The available documented treatment data also suggest that early anti-schistosomal treatment may prevent the fertility-damaging effects of urogenital schistosomiasis, and lend support for programs that provide universal treatment of children in *S*. *haematobium*-endemic regions.

## Author summary

Infertility is an unwelcome complication of many infectious diseases. In sub-Saharan Africa, where women experience the highest rates of subfertility in the world, the helminthic parasite *Schistosoma haematobium*, is also highly prevalent. Chronic and repeated infections with *S*. *haematobium* cause inflammation of the pelvic organs and kidneys, a condition known as urogenital schistosomiasis. Past studies have statistically linked past and present *S*. *haematobium* infection to the risk of infection-associated female genital lesions and with risk for subfertility in adulthood. The present study used in-depth interviews to document reproductive health histories of 162 women, aged 15 to 62, residing in an *S*. *haematobium*-endemic area of coastal Kenya, in order to examine the association of their earlier anti-schistosomal treatment with their fertility patterns. A self-reported history of past treatment was not associated with lower odds of infertility. However, among 61 with verified treatments given during previous local campaigns, it was found that women who were treated before age 21 experienced significantly fewer period of subfertility during their adult life. The findings suggest that anti-schistosomal treatment during childhood may prevent the fertility-damaging effects of urogenital schistosomiasis. They also lend support for programs that provide universal treatment of children in *S*. *haematobium*-endemic regions.

## Introduction

Previous studies have identified a so-called “infertility belt” across central sub-Saharan Africa [1–6]. While worldwide infertility surveys generally find that 8 to 12% of couples experience sub-fertility or infertility, parts of sub-Saharan Africa report infertility rates of up to 30% [2, 3, 5, 6]. Infertility in these areas is associated with high rates of pelvic infections that cause damage to the female and male reproductive organs [1, 2]. In most cases, inflammation-mediated damage results in secondary infertility, which is infertility after a woman has already had a live birth, rather than primary infertility, which is the inability to have a live birth [5, 6]. Past research has documented the very significant social and psychological impact of infertility on women in low-resource countries and communities [4, 7–11]. Infertility can be particularly devastating for women in communities in which motherhood is a key aspect of female gender identity [3, 4, 9]. Inhorn [7–9] has described the experience of infertility for women in these communities as a unique form of gendered suffering, which can exclude women from important social roles.

Urogenital schistosomiasis results from infection with the trematode parasite, *Schistosoma haematobium*. It is the most prevalent form of human schistosomiasis [12] and is found in 53 countries [13]. For women living in *S*. *haematobium* endemic regions, the manifestations of Female Genital Schistosomiasis (FGS) have been reported among 30 to 70% of long-term residents [14, 15]. Although traditionally described as the ‘urinary’ form of schistosomiasis, *S*. *haematobium* actually infects both the urinary and the genital systems, creating potential reductions in fertility for both men and women due to chronic inflammation and scar formation in the reproductive tract [14, 16–20].

As part of a larger, qualitative anthropological survey of factors related to sub-fertility in rural Kenya, this pilot study investigated the potential association of prior *S*. *haematobium* infection and of individual-level treatment history with subfertility rates among women from Msambweni, Kenya, an area endemic for urogenital schistosomiasis [10, 11].

## Methods

### Study design

The interview-based cross-sectional study was developed to explore both self-reported fertility patterns and reproductive health practice and attitudes in an area endemic for urogenital schistosomiasis. Full results for other aspects of the project will be reported in a separate publication.

### Ethical approval

Ethical approval and oversight for this study was jointly provided by the Institutional Review Board of the University Hospital Case Medical Center of Cleveland (Protocol 11-07-45) and by the Ethical Review Committee of the Kenya Medical Research Institute (KEMRI) (Non-SSC Protocol 087). All women residents from the selected study village were eligible for inclusion as participants in the study if they met the inclusion criteria outlined below. All participants provided written informed consent or oral assent to be interviewed. A parent or guardian of women participants under the age of 21 provided informed consent on their behalf.

### Study setting

For an exploratory evaluation of the impact of *S*. *haemetobium* infection and of anti-schistosomal treatment, health histories of women in the study area were collected via oral interviews and matched to infection and treatment data from prior community-based treatment studies of this location [10, 11]. After determining the prevalence of subfertility in the study population, we used the linked childhood infection and treatment data to determine possible associations between current or past *S*. *haematobium* infection and subfertility. Interviews for this cross-sectional study were conducted during June and July of 2012 in a rural community in the Msambweni location of Kwale County, Kenya. Previous surveys conducted between 1984 and 2009 had established *S*. *haematobium* prevalence rates of 60–85% among school age children in the village participating in this study [21, 22], indicating an area of high endemicity by WHO criteria. The community had previously participated in collaborative studies of the disease burden of schistosomiasis and of the impact of anti-schistosomal treatment on year-to-year infection status.

Diagnostic and treatment data for study participants included in the current analysis were obtained from established computer databases of these 1984–2009 studies, collectively known as the Msambweni Project [10, 11, 21, 22]. From 1984–1992, local school age children were screened and treated for *S*. *haematobium* infection annually in a schools-based operational research program jointly sponsored by The Kenyan Ministry of Health and Case Western Reserve University [11, 21]. Enrolled children were monitored through regular school and household censuses, with documentation of their yearly infection and treatment status during that period. Later, during the period 2000 to 2009, Nganja village residents over the age of 5, including adults, participated in a periodic house-to house infection screening and community-based treatment program for urogenital schistosomiasis as part of an extended study of the ecology of *S*. *haematobium* transmission [10, 22].

### Study participants

Women currently residing in Nganja village were eligible for inclusion in this study if they met at least one of three inclusion criteria: (1) they had ever been married (defined as a co-residing sexual union [23]), (2) they were currently pregnant, or (3) they had given birth to at least one child. A total of 162 women were interviewed, encompassing all women identified as meeting the inclusion criteria by study demographers, who were local community members participating in the research team.

### Outcomes and covariates evaluated

For this study, the primary outcome, subfertility, was treated as a dichotomous variable and its presence or absence was determined based on the results of extended reproductive health interviews with participants. Subfertility was defined as the absence of a live birth for a woman between the ages of 15 and 45, who was in a sexual union and not using contraception for at least 5 years, according to the definition of Mascarenhas and colleagues [24], and/or a woman reporting a period of over one year without a pregnancy with regular, unprotected sexual intercourse, per the WHO standardized definition [25].

The experience of any *S*. *haematobium* infection in childhood was captured as a categorical variable, either: (1) as *confirmed infection* for those women with records of positive *S*. *haematobium* egg detection in the urine in previous surveys, (2) as *likely infection* for women who self-reported childhood infection in the study interview, or for women raised in high prevalence *S*. *haematobium-*endemic areas as indicated by village of birth and/or primary school attended, or (3) as *unlikely infection* for women with consistently negative test records in databases from previous surveys, or women who were raised in low prevalence areas as indicated by village of birth and/or primary school. Local prevalences of *S*. *haematobium* infection were based on earlier school-and community-based surveys conducted in the region [26–30]. Based on urine screening, infection prevalence over 10% was considered moderate-to-high local *S*. *haematobium* prevalence, with nearly all participants from such a region likely to have been infected at some point during childhood [29, 31].

Anti-schistosomal treatment effect was analyzed based on either self-reported or documented prior treatment, and was scored as a dichotomous variable either as confirmed or likely infection with treatment *or* confirmed or likely infection without treatment. Age at first treatment was also determined based on verified data from previous community treatment studies.

### Data sources and measurement

This study’s data collection questionnaire was developed by the Anthropology Department at Case Western Reserve University. At the study site, the questionnaire was translated into the local Kidigo dialect by two fieldworkers, each with over 5 years of experience in local interviewing. The questionnaire was then piloted with three community members who were not included in the study, and subsequent clarifications were made, resulting in the final version of the questionnaire used for all women in the study (see S1 Text). The fieldworkers conducted all of the interviews, and responses were recorded in a standard form (see S2 Text) from which data were entered into a computer database. Whenever possible, participants’ and their children’s ages were verified via written records (e.g. school or clinic records) or confirmation by other family members.

In documenting past experience with laboratory-confirmed urinary *S*. *haematobium* infection and with anti-schistosomal treatment, participants were matched to their previous Msambweni study ID records based on their full names, their approximate ages, their parents’ and siblings’ family names, and the project’s school treatment records, cross-indexed to childhood household census numbers [11, 32, 33].

### Statistical methods

All quantitative data for this study were entered into a central database in both Microsoft Excel and SPSS data files, and analyzed using SPSS v.21 (IBM Corp., Armonk, NY). Qualitative data was analyzed using Dedoose software (available at http://www.dedoose.com).

Chi square analysis and Fisher’s exact test were used to determine significant associations between previous *Schistosoma* infection and subfertility. These methods were also used to analyze the relationship between subfertility and prior anti-schistosome treatment or subject age category at the time of first treatment. Logistic regression was then used to determine association between age at first treatment (as a continuous variable), the number of reported treatments, current age at the time of interview, and the relative odds of experiencing subfertility.

## Results

**Fig 1:**
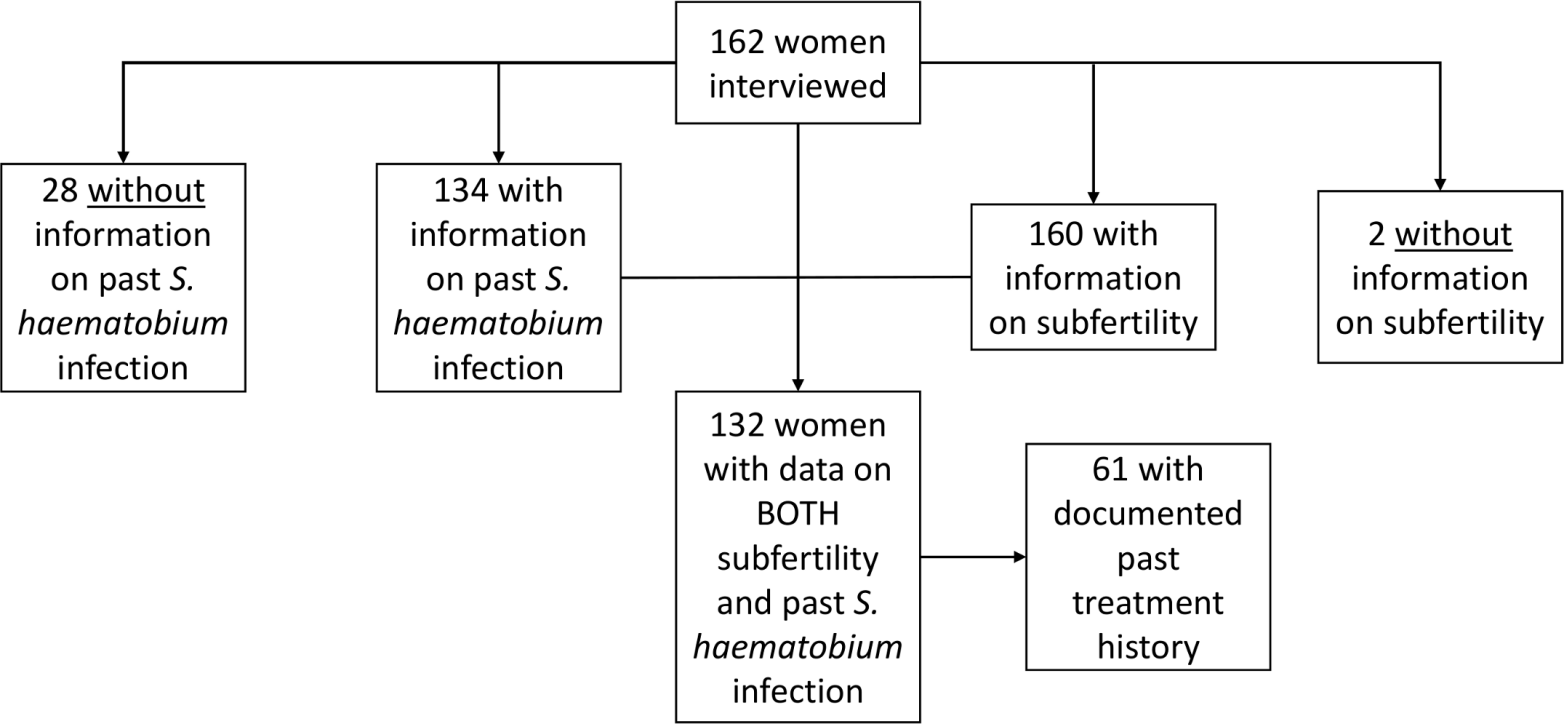
Flow diagram of study subject subgroups analyses. The boxes indicate the number of women having information available on subfertility, on past *S*. *haematobium* infection, and on documented past treatment, respectively.

162 participants were interviewed for this study. There were sufficient fertility history data for determining subfertility rates for 160 of these participants, and there were self-reported or documented *Schistosoma* infection data for 134. Fig 1 indicates the subjects available for each phase on study analysis. Overall, there were 132 participants for whom both subfertility and past infection history could be determined. Among that group, 61 participants also had past treatment records available from school- and community-based studies of anti-*S*. *haematobium* mass drug administration in the region [10, 11, 21, 32].

The basic fertility patterns of the entire study population of women in Nganja Village, Kwale County, Kenya are presented in Table 1. The population ranged in age from 15 to 62 years, with a mean of 12.5 (SD 8.6) years of exposure to pregnancy (defined as cohabitating in a sexual relationship without the use of contraception). The mean parity was 3.9, less than the mean desired number of children, which was 5.1. The mean abortus rate was 0.5, and the percent of all pregnancies ending in miscarriage or stillbirth was 12%.

**Table 1:** Description of fertility characteristics for the overall study population (N = 162).

Characteristic	Mean ± SD	Range
Age in years at time of interview	38.6 ± 13.1	15–62
Years of exposure to pregnancy	12.5 ± 8.6	1–30
Gravidity (times pregnant)	4.1 ± 2.6	0–12
Parity (births after 20 weeks)	3.9 ± 2.4	0–10
Desired number of children	5.1 ± 2.3	2–20
Abortus (pregnancy not resulting in live birth)	0.5 ± 0.8	0–4
Percent of all pregnancies ending in miscarriage or stillbirth	12% (10–15%)^†^	---

^†^95% Confidence Interval

### Subfertility

The presence or absence of subfertility could be ascertained for 160 women (99% of the study population). Overall, the rate of subfertility was 44%. Primary infertility was much less common than secondary infertility (Table 2). On average, these affected women experienced 9.6 (SD 5.6) years of subfertility.

**Table 2:** Overall group prevalence of subfertility and past *S*. *haematobium* infection status.

Status	Source	Count/Total	Percent affected (CI_95%_)
Subfertility	Interview	71/160	44% (37–52%)
Primary infertility	Interview	3/160	2% (0.3–5%)
Secondary infertility	Interview	68/160	42% (35–51%)
Confirmed infection	Previous databases	67/162	41% (34–49%)
Probable infection	Health history, village of origin or primary school	57/162	35% (28–43%)
Unlikely infection	Previous databases, village of origin or primary school	10/162	6% (3–11%)
Unknown infection status	---	28/162	17% (12–24%)

Previous *Schistosoma* infection was determined for 134 participants in the study, using a combination of; i) self-reported health histories from the women, ii) recorded test data from prior studies, and iii) *S*. *haematobium* prevalence data for their villages of origin and/or schools attended. The majority of participants (76%) had either a confirmed or probable previous infection. Infection was unlikely in 6% of participants. Infection status could not be determined for 28 (17%) of the 162 women interviewed (Table 2 and Fig 1).

### Association between infection status and subfertility

We next analyzed the relationship between infection and subfertility for the 132 participants with complete data on infection status and subfertility (Table 3). The highest prevalence of subfertility was found for women in the ‘unlikely infection’ category; however, this category included only 10 participants.

**Table 3:** Subfertility prevalence according to past *S*. *haematobium* infection status (N = 132).

Status	N for Category	Subfertility	Percent affected (CI_95%_)
Confirmed infection	66	25	38% (26–51%)
Probable infection	56	25	45% (31–59%)
Unlikely infection	10	8	80% (44–97%)
**Total**	132	58	44% (35–53%)*

*χ^2^ = 6.27, *P* = 0.0435 for significant difference among groups.

### Association between anti-schistosomal treatment status and subfertility

We next examined the relationship between past treatment for *S*. *haematobium* and the odds of present or past subfertility (Table 4). When subject self-report of treatment and documented study treatments were considered together, we found no significant association between such treatment histories, in general, with subfertility.

**Table 4:** Subfertility among women with positive infection status according to their treatment history (N = 122).

		Subfertility		No Subfertility	
Status	N	N (%)	CI_95%_	N (%)	CI_95%_
**Confirmed or probable infection with self-reported or documented treatment**	82	34 (42%)	31–53%	48 (58%)	47%, 69%
**Confirmed or probable infection without treatment**	40	16 (40%)	25–57%	24 (60%)	43–75%
**Total**	122	50 (41%)*	32–50%	72 (59%)	50–68%

*χ^2^ = 0.2, difference not significant

Focusing on the documented treatment records that were available for a subset of 61 study women, we next analyzed their experience, specifically the effect of single vs. multiple childhood treatments and of the age at first treatment on odds of having experienced subfertility.

For these women, the number of times treated ranged from 1 to 7 (median = 1, inter quartile range (IQR) = 1 to 2), the median age at first treatment had been 13 years (IQR: 9 to 39; range: 4–50 years) and median age at last treatment was 17 years (IQR: 11 to 39; range: 4–50 years). By logistic regression analysis, although the odds of subfertility were lower with a greater number of reported lifetime treatments, this effect was not statistically significant (Table 5**).** However, we did observe a highly significant positive association (P = 0.001) between having an older age at the time of first anti-schistosomal treatment and the odds of experiencing subfertility (Table 5). These estimates indicated that the odds of subfertility were increased approximately 6% for each additional year’s delay in treatment.

**Table 5:** Effects of multiplicity and timing of documented anti-schistosomal treatment on the odds for subfertility (N = 61).

Covariate	Odds Ratio	CI_95%_	P value
Number of treatments reported	0.743	0.445, 1.240	0.255
Age at first treatment	1.065	1.044, 1.089	0.001

**Fig 2:**
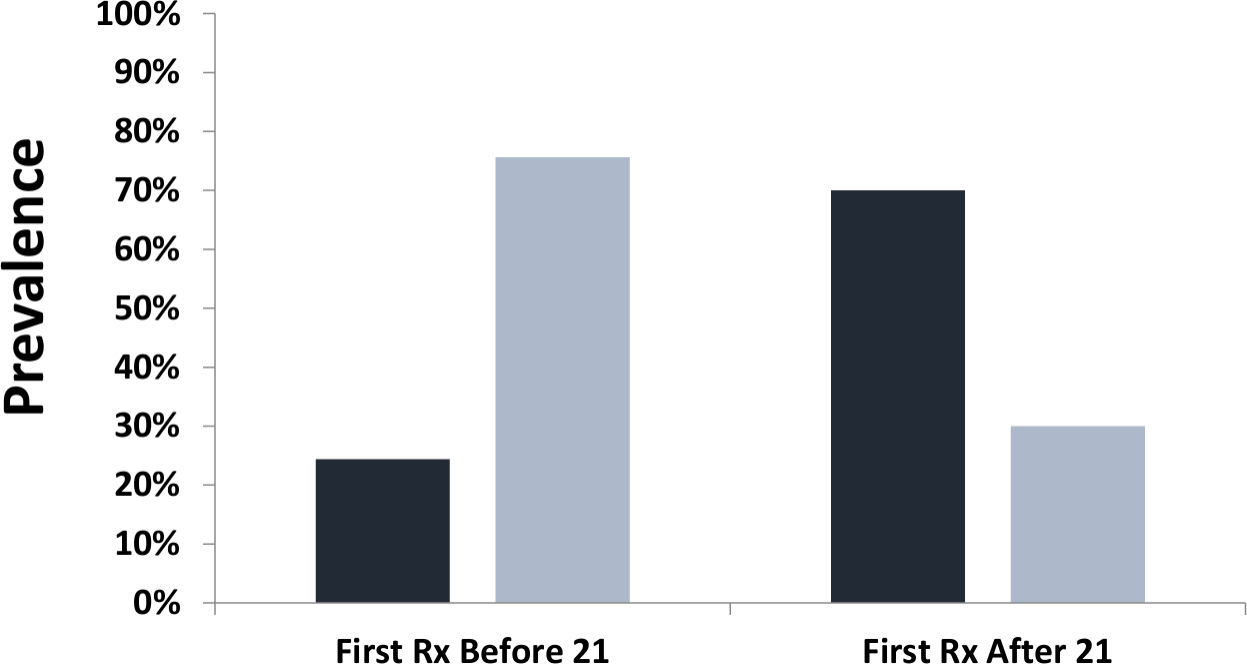
Subfertility (dark bars) vs. normal fertility (light bars) according to the timing of first anti-schistosomal treatment. The association between earlier anti-schistosomal treatment and fertility status is indicated according to whether the study participant (N = 61) had documented treatment before age 21 or after. Abbreviation: Rx indicates drug treatment.

Because current WHO treatment guidelines are focused mainly on delivery of mass drug administration targeted to school age children, we next looked specifically at the effect of receiving the first anti-schistosomal treatment before or after age 21 (Fig 2). Among the 61 women with documented treatment histories, for those who received treatment before age 21 (N = 41), ten women or 24% experienced sub-fertility, whereas among women who received their first treatment only at or after age 21 (N = 20), 14, or 70% had experienced subfertility (P = 0.001 by Fisher’s exact test).

Subfertility rates were compared between women with documented treatment records who had received their first schistosomiasis treatment before age 21 (N = 41) and those who had received it at age 21 or after (N = 20). Subfertility rates were significantly higher in the population of women receiving treatment after age 21 (OR = 7.2, CI_95%_ 2.2, 24; P = 0.001).

In considering potential confounding factors for the fertility effect observed in our analysis, we noted that those women who had been treated before age 21 were significantly younger (mean ± SD age: 31.6 ± 7.0 vs. 52.0 ± 9.3 years, *P* < 0.001) and had had more years of schooling (mean years of school 8.2 ± 3.1 vs. 4.5 ± 4.0, *P* = 0.001) than women who had been treated only after age 21. As a potential source for variation, younger age also meant that, as a group, the women treated before age 21 had had fewer years of exposure to pregnancy than those treated after age 21 (9.0 ± 6.7 vs. 20.4 ± 7.2 years, P < 0.001) by the time of their interview for this study. When adjusted for present age at the time of interview, the odds of having experienced sub-fertility remained higher when a woman had not received treatment before age 21 (aOR = 3.4, CI_95%_ 0.6, 21), although small sample size limited the ability to show statistical significance for this observed difference (*P* = 0.18).

## Discussion

This study explored *S*. *haematobium* infection as a possible cause of high subfertility rates in women in endemic areas of coastal Kenya. While we were unable to establish a significant link between subfertility and a reported history of *S*. *haematobium* infection *per se*, this may have been due to the low numbers of unexposed women in our comparison, ‘probably uninfected’ population. We did not identify a significant association between subfertility and a reported history of having received anti-schistosomal treatment at any point. However, when we were able to obtain documented treatment records from past community studies, the age at *first* anti-schistosomal treatment proved to be significantly related to the lifetime odds of experiencing subfertility. Specifically, there was a highly significant difference in subfertility rates between women who received their first documented treatment before age 21 versus an older age, with lower subfertility rates noted among women who had received treatment earlier in their lives.

There were limitations to our study. The study size was restricted by the number of participants who were eligible in the targeted study area (162 women), although it is notable that all eligible women agreed to participate, likely due to the leadership of the local recruitment and interview teams. The study is also limited by the amount of data available within previous treatment databases. This, in turn, limited our ability to further adjust for potential confounding factors. Recall bias over several decades may have affected both the accuracy of fertility and of self-reported infection/treatment data as related by the participating women during their interviews. A time cohort effect may have also had an impact on available results, as available treatment records essentially divided the participants into those from school-based targeted-mass treatment studies in 1984–1992 [11, 28] from those in community-based studies in 2000, 2003, and 2009 [10, 32]. Women treated as children between 1984 and 1992 received more intensive, school based annual screening and treatment during that era. Their current ages would range from 25–43. Women outside that current age range could have only been treated after 2000, and then only once or twice. This difference could have played a role in the age effects we have observed. Another limitation of this study was that the high regional prevalence of *S*. *haematobium* infections limited our ability to link self-reported *S*. *haematobium* infection with subfertility. Many low intensity infections may be minimally symptomatic and even egg-negative on a single daily urine examination [15, 34]. The low number of truly unexposed/uninfected participant women meant that the study was underpowered to demonstrate a clear effect of reported past infection or exposure on local subfertility. Because of fiscal constraints, the study was limited to the adult female population of Nganja village, and because we could not predict the number of participants who would have documented treatment data, no a priori sample size determination was done. As such, the 61 participants used in this phase of the analysis must be considered a convenience sample, and the results of this pilot study should be taken with appropriate caution.

By contrast, a strength of the study was that previous longitudinal datasets provided individual-level information on treatment, which allowed us to compare specific age-dependent effects of prior anti-schistosomal treatment on lifetime chances of subfertility. Whereas other studies have provided evidence that residence in a *S*. *haematobium*-endemic area is associated with risk of subfertility [16], and that there is a correlation between one’s age of exposure to treatment campaigns and later risk of FGS [15], our analysis is the first to use documented treatment records to affirm an individual-level connection between earlier childhood treatment and lower odds of experiencing subfertility.

Overall, our results suggest that early treatment can prevent the fertility-damaging effects of urogenital schistosomiasis and reduce rates of subfertility in later adulthood. A study in Zimbabwe has shown that exposure to anti-schistosomal treatment campaigns before age 20 is significantly associated with a reduced rate of genital pathology related to schistosomiasis in adulthood [15]. In particular, there was a demonstrated lower prevalence of contact bleeding and sandy patches in the genital tracts of women exposed to earlier treatment [15]. Taken together, our combined studies’ evidence indicates that urogenital schistosomiasis caused by *S*. *haematobium* infection causes pathology that reduces fertility, but this morbidity may be halted or prevented with early treatment. Independent surveys are now in progress in our study region, Kwale County, to document the frequency of FGS pathology in local *S*. *haematobium*-endemic communities undergoing treatment. Those findings are expected to provide supporting evidence by documenting the frequency of anatomical FGS findings in the local population of adult women, including our study area.

Although we specifically focused on subfertility outcomes, the lifelong benefits of anti-schistosomal treatment likely extend across multiple areas of health. In addition to its risk of infertility, female genital schistosomiasis is a risk factor for ectopic pregnancy [35–37], and damage to genital tissue due to schistosomiasis has been associated with increased odds of HIV infection or other sexually transmitted infections [14, 38–40]. As a whole, the current literature suggests that regular anti-schistosomal treatment during childhood and early adulthood should have a significant impact on multiple infection-associated chronic morbidity outcomes, including subfertility and, thus, we recommend the implementation treatment programs for all children in *S*. *haematobium*-endemic areas with moderate to high risk of infection. Because infertility is particularly disabling for women in communities where motherhood is an important aspect of female gender identity [3, 4, 9], FGS and reproductive health should be clear priorities for morbidity control and prevention.

## Supporting information

S1 Table STROBE checklist. (DOC)

S1 Text Subject questionnaire: English and Kiswahili versions. (DOCX)

S2 Text Response recording form. (DOCX)

S3 Text Access to study data. (DOCX)
