# Supplementary material for: Cross-sectional interview study of fertility, pregnancy, and urogenital schistosomiasis in coastal Kenya: Documented treatment in childhood is associated with reduced odds of subfertility among adult women
Source: PLoS Negl Trop Dis. 2017 Nov 27;11(11):e0006101. doi: 10.1371/journal.pntd.0006101 (PMC5720807; doi:10.1371/journal.pntd.0006101)
Supplement: S1 Table — (DOC) [file pntd.0006101.s001.doc]

STROBE Statement—Checklist of items that should be included in reports of ***cross-sectional studies***

|  | Item No | Recommendation |
| --- | --- | --- |
| **Title and abstract** | 1 | (*a*) Indicate the study’s design with a commonly used term in the title or the abstract Line 1 |
|  |  | (*b*) Provide in the abstract an informative and balanced summary of what was done and what was found Lines 24-43 |
| Introduction | | |
| Background/rationale | 2 | Explain the scientific background and rationale for the investigation being reported  Lines 64-83 |
| Objectives | 3 | State specific objectives, including any prespecified hypotheses Lines 88-91 |
| Methods | | |
| Study design | 4 | Present key elements of study design early in the paper Lines 94-96 |
| Setting | 5 | Describe the setting, locations, and relevant dates, including periods of recruitment, exposure, follow-up, and data collection Lines 103-111 |
| Participants | 6 | (*a*) Give the eligibility criteria, and the sources and methods of selection of participants Lines 1123-119 |
| Variables | 7 | Clearly define all outcomes, exposures, predictors, potential confounders, and effect modifiers. Give diagnostic criteria, if applicable Lines 120-143 |
| Data sources/ measurement | 8* | For each variable of interest, give sources of data and details of methods of assessment (measurement). Describe comparability of assessment methods if there is more than one group Lines 144-158 |
| Bias | 9 | Describe any efforts to address potential sources of bias Line 166-168 |
| Study size | 10 | Explain how the study size was arrived at Lines 115-117 |
| Quantitative variables | 11 | Explain how quantitative variables were handled in the analyses. If applicable, describe which groupings were chosen and why Lines 120-168 |
| Statistical methods | 12 | (*a*) Describe all statistical methods, including those used to control for confounding Lines 159-168 |
|  |  | (*b*) Describe any methods used to examine subgroups and interactions Lines 166-168 |
|  |  | (*c*) Explain how missing data were addressed Lines 171-177 |
|  |  | (*d*) If applicable, describe analytical methods taking account of sampling strategy |
|  |  | (*e*) Describe any sensitivity analyses- N/A |
| Results | | |
| Participants | 13* | (a) Report numbers of individuals at each stage of study—eg numbers potentially eligible, examined for eligibility, confirmed eligible, included in the study, completing follow-up, and analysed Figure 1 |
|  |  | (b) Give reasons for non-participation at each stage Lines 171-178 and Figure 1 |
|  |  | (c) Consider use of a flow diagram- Figure 1 |
| Descriptive data | 14* | (a) Give characteristics of study participants (eg demographic, clinical, social) and information on exposures and potential confounders Lines 182-187, Table 1. |
|  |  | (b) Indicate number of participants with missing data for each variable of interest Lines 171-178 |
| Outcome data | 15* | Report numbers of outcome events or summary measures Tables 1 and 2 |
| Main results | 16 | (*a*) Give unadjusted estimates and, if applicable, confounder-adjusted estimates and their precision (eg, 95% confidence interval). Make clear which confounders were adjusted for and why they were included Tables 4 and 5, Lines 253-263 |
|  |  | (*b*) Report category boundaries when continuous variables were categorized |
|  |  | (*c*) If relevant, consider translating estimates of relative risk into absolute risk for a meaningful time period- N/A |
| Other analyses | 17 | Report other analyses done—eg analyses of subgroups and interactions, and sensitivity analyses N/A |
| Discussion | | |
| Key results | 18 | Summarise key results with reference to study objectives Lines 266-276 |
| Limitations | 19 | Discuss limitations of the study, taking into account sources of potential bias or imprecision. Discuss both direction and magnitude of any potential bias Lines 277-291; |
| Interpretation | 20 | Give a cautious overall interpretation of results considering objectives, limitations, multiplicity of analyses, results from similar studies, and other relevant evidence Lines 292-299 |
| Generalisability | 21 | Discuss the generalisability (external validity) of the study results Lines 300-321 |
| Other information | | |
| Funding | 22 | Give the source of funding and the role of the funders for the present study and, if applicable, for the original study on which the present article is based This is loaded in the PNTD site and will appear in the published article after the author list. |

*Give information separately for exposed and unexposed groups.

**Note:** An Explanation and Elaboration article discusses each checklist item and gives methodological background and published examples of transparent reporting. The STROBE checklist is best used in conjunction with this article (freely available on the Web sites of PLoS Medicine at http://www.plosmedicine.org/, Annals of Internal Medicine at http://www.annals.org/, and Epidemiology at http://www.epidem.com/). Information on the STROBE Initiative is available at www.strobe-statement.org.
