# Supplementary material for: Cross-sectional interview study of fertility, pregnancy, and urogenital schistosomiasis in coastal Kenya: Documented treatment in childhood is associated with reduced odds of subfertility among adult women
Source: PLoS Negl Trop Dis. 2017 Nov 27;11(11):e0006101. doi: 10.1371/journal.pntd.0006101 (PMC5720807; doi:10.1371/journal.pntd.0006101)
Supplement: S1 Text — (DOCX) [file pntd.0006101.s002.docx]

**Appendix 1.1** – Study Questionnaire (English Version)

**Introduction and Consent:**

This is Sarah/Laura. She is a student coming from America to do research on reproductive health of women in Nganja. Today we are here to ask you some questions about you and your family in order to link this study to previous research in Nganja. We are trying to understand how childhood health can impact the pregnancies and children a woman has as an adult. You will be asked about yourself and your family members’ basic information, your marriages, your pregnancies, and some general questions about pregnancy. Whatever answers you give will help us in our research. If you are uncomfortable with or choose not to answer any question it is okay to skip the question. Choosing not to answer the questionnaire or any of these questions will not affect your participation in other research projects. Your answers will be kept private and not be shared with others in your community. Your name and other identifying information will not be used in any writings or presentations of this data. Are you willing to answer these questions?

**Basic Biographical Information:**

What is your name?

What year were you born?

In what village were you born?

Did you go to school? What schools did you attend?

What was the highest level of school you completed?

Are you or have you ever been married? What is your most recent husband’s name?

What year was he born?

In what village was he born?

Did he go to school? What schools did he attend?

What was the highest level of school he completed?

(repeat questions for all previous husbands)

What is your mother’s name?

What year was she born?

In what village was she born?

Did she go to school? What schools did she attend?

What was the highest level of school she completed?

What is your father’s name?

What year was he born?

In what village was he born?

Did he go to school? What schools did he attend?

What was the highest level of school he completed?

What are your siblings’ names?

For each sibling:

What year was he or she born?

In what village was he or she born?

Did he or she go to school? What schools did he or she attend?

What was the highest level of school he or she completed?

Does this sibling have the same mother?

**Information about Husbands and Fathers of Children:**

Can you tell me about your first husband?

What was his name?

When were you married?

When did your marriage end?

How many children does (name) have?

(Repeat for each husband/partner)

**Childhood Health**

Did you have any major sicknesses when you were a child?

For each illness:

What age were you?

What was done to treat the illness?

Where were treatments (if formal healthcare facility)?

**Avoiding Pregnancy:**

What have you done to avoid pregnancy throughout your life?

For each method:

How long did you/have you used this method? (Ask if dates are known or between which pregnancies.)

Where did you obtain this method?

**Pregnancies:**

Tell me about your first pregnancy.

What was the date of delivery? (Can consult clinic card or use other reference point such as age mates).

How many months did this pregnancy last?

What is the current health status of this child? (Include here if miscarriage)

What is/was the name of this child?

Where did you give birth?

Who helped you to give birth?

Tell me about your next pregnancy. (Repeat until all pregnancies have been described.)

What was the date of delivery?

What is/was the name of this child?

How many months did this pregnancy last?

Where did you give birth?

Who helped you to give birth?

What is the current health status of this child? (Include here if miscarriage)

After all pregnancies have been described: Was (name) the father of all of these children? If says no, who is the father of (names of children not named)? (Repeat questions about each father.)

**Questions about Becoming Pregnant:**

Have you had periods of time when you wanted to be pregnant but were not?

When were these periods? (e.g. between which pregnancies)

How long did each of these periods last?

If you were ever concerned that you were not becoming pregnant, what did you do to help become pregnant?

How many children is a good number for a woman to have?

What can a girl do to make sure that she is able to have healthy pregnancies and babies when she is a woman?

What does a woman do to prepare for motherhood before pregnancy?

What does a woman do to prepare for motherhood during pregnancy?

What precautions during a pregnancy should a woman take to produce a healthy baby?

Where are you planning to give birth the next time you are pregnant?

Who would you like to attend the delivery with you?

**Appendix 1.2** – Study Questionnaire (Kiswahili Version)

**Wtangulizi na Idhini**

Hawa ni Laura/Sarah wanafunzi kutoka USA. Wanekiya kufanja utafiti kulwsiane na afya ya uzazi kwa wanawake wa Nganja. Tuko hapa Kuwahiza mawah yanayo husu nyinyi na famii zenu. Kuunganisha ha utafuti uliyopita. Tunajaribu kutaka krojuwa vipi afya yako ya utotoni inawega kuathiri mimba na watoto utakopofikia utu uzima. Utaulizwa maswali kuhusu wewe na jamii yako yana historia yako yohusiana na maelezo muhimu ya (le miaka ya kuzaliwa/shule/kijiji), ndoa uja uzito, na maswahi kuhusu uja uzito kwa jumla. Majibu yako yatasaidia kwenye utafiti wetu. Unao uamuzi wa kutujibu kati ya maswali utakayo ulizwa. Kama huridhiki nalo. Kwa uamuzi wako wa kutotakea kujibu maswali hakuta thiri na kujiunga na miradi ya utafiti. Marjibu yako yafa hifadhiwa ki siri bila ya kujuhi kana na mtu mwengine wa kijiji chako. Jina na maelezo kukuhusu hayataonyeshwa kweiye karatasi ya maelezo. Je ukotayari kujibu maswali?

**Maelezo Muhimu**

Jina lako ni nani?

Ulazaliwa mwaka gani?

Ulizahiwa kijiji gani?

Uliwahi kisoma/ulienda shule gani?

Uhifikia kiwango dia elimu?

Uliwahi kwolewa ama umeolewa?

Mume wako wa sasa anaifwaje?

Alizaliwa mwaka gani?

Alizaliwa kijiji gani?

Aliwali kusoma?

Alienda shule gani.

Alifikia kiwango gani cha elimu?

(Rejela maswali ya bwana zake wa kabla/nyuma).

Mamako anitwaje?

Alizah’wa mwaka gani?

Alizah’wa kijiji gani?

Je aliwahi kwenda shule.

Je alienda shule gani?

Alifikia kiwango gani cha elimu?

Babako anaitwaje?

Ahizah’wa mwaka gani?

Ahizah’wa kijiji gani?

Je aliwahi kwenda shule.

Alienda shule gani?

Alifikia kiwango gani cha elimu?

Ni yapi ya ndugu na dada zako?

Kwa kilandugu?

Alizah’wa mwaka gani?

Alizah’wa kijiji gani?

Je aliwahi kwenda shule?

Je alienda shule gani?

Alifikia kiwango gani cha elimu.

Je ndgugu huyu wlizaliwa naye tumbo moja?

**Maelezo kuhusu mabwana na baba wa watoto wao:**

Je Unaweza kuni ambia bwana wako wa kwanza?

Alikuwa anaitwaje?

Mlioana lini?

Ndoa yenu ihisha lini?

Je ana watoto wangapi?

(rejelea kwa kila bwana).

**Afya ya utotoni**

Je uliwahi kungua uhipokuwa mtoto?

Kwa ila ugonjwa:

Ulikuwa na umri gani?

Ulipata huduma gani ya kuutibu?

Wapi ulitibiwa (kama ni kweye vitro vya afya)

**Kuepuka uja-uzito**

Ni njia zipi ulizozitumia kuepukana na uja-uzito maishani mwako?

Kwa kila njiai:

Ulitumia kwa muda gani (uliza ikiwa tavehe yajulikana ama kati ya mimba)

Ulipata wapi jia hii?

**Uja uzito**

Nielezee kuhusu mimba yao ya kwanza.

Ulizaa taveho ngapi (unaeza kuitisha kadi ya klimiki ama ukumbusho wa mfano wa rika lake).

Je uliibeba kwa mizi mingapi?

Je kwa sasa ah yake ja afya iko vipi? (ongezea kama ilimwagika).

Je anaitwaje/aliitwaje?

Ulimzalia wapi?

Ni nami aliye ku alisha?

Nielezee kuhusu mimba nyenginezo (rejelea maelezo ya mimba zote).

Baada ya maelero ya mimba zote kutolewa: Je (jina) alikuwa baba wa watoto wote?

Kama la, ni nani baba ya (majima ya watoto ambao hawakutujwa)?

(Rejelea maswali kuhuju kila baba).

**Maswali kuhusu kuwa mja-mzito**

Kuna wakati uliotaka kuwa mja-mzito lakimi haikuwa?

Ilikuwa wakati gani (e.g. Katika ya mimba zipi).

Kwa kila shida ilichukuwa muda gani?

Kama uliwahi kupati kana ya kuwa hutopata uja uzito, ni haitia gani ulichukuwa kuku wezesha kupata uja uzito?

Ni idadi gani ya watoto nzuri kwa mama anayahita-jika kuwa nayo?

Ni njia gani mschina anaweza kufanya ih’kumahakikishia anaweza kupata mimba yenye afya na watoto wakati akifiki umri wa utu-uzima?

Ni maandalizi gani mama anapawa kufanya kabla hajapata uja-uzito?

Ni maandalizi gani mama anapaswa kufanya wakati ana uja-uzito?

Ni tahadahari zipi mama mja mzito anapaswa kuzichukua ili aweze kuzaa mtoto mwenge afye?

Umejpangia kuzaliawapi kwa uja uzilo wa baadaye?

Ni nani ungependa akahudhurie siku ya kujifungua.
