# Supplementary material for: Cross-sectional interview study of fertility, pregnancy, and urogenital schistosomiasis in coastal Kenya: Documented treatment in childhood is associated with reduced odds of subfertility among adult women
Source: PLoS Negl Trop Dis. 2017 Nov 27;11(11):e0006101. doi: 10.1371/journal.pntd.0006101 (PMC5720807; doi:10.1371/journal.pntd.0006101)
Supplement: S2 Text — (DOCX) [file pntd.0006101.s003.docx]

Appendix 2 – Study Response Form

**Reproductive Health Interview – for all women who have children, are currently pregnant, or who are married**

**Date**:__________________________ **Time**: ________________________ **Village**: __________________________________

**Names of Interviewers**: ____________________________ and ________________________________

**Interviewee identification #**: ___________________________________

**Six-digit ID #**:____________________________________

**Basic Biographical Information**

|  | Name | Year of birth | Village of birth | Name of school attended | Highest year in school completed | Same mother (sibling) |
| --- | --- | --- | --- | --- | --- | --- |
| Woman (ego) |  |  |  |  |  |  |
| Husband* |  |  |  |  |  |  |
| Husband |  |  |  |  |  |  |
| Husband |  |  |  |  |  |  |
| Husband |  |  |  |  |  |  |
| Mother |  |  |  |  |  |  |
| Father |  |  |  |  |  |  |
| Sibling (m or f) |  |  |  |  |  |  |
| Sibling (m or f) |  |  |  |  |  |  |
| Sibling (m or f) |  |  |  |  |  |  |
| Sibling (m or f) |  |  |  |  |  |  |
| Sibling (m or f) |  |  |  |  |  |  |
| Sibling (m or f) |  |  |  |  |  |  |
| Sibling (m or f) |  |  |  |  |  |  |
| Sibling (m or f) |  |  |  |  |  |  |
| Sibling (m or f) |  |  |  |  |  |  |
| Sibling (m or f) |  |  |  |  |  |  |
| Sibling (m or f) |  |  |  |  |  |  |
| Sibling (m or f) |  |  |  |  |  |  |
| Sibling (m or f) |  |  |  |  |  |  |
| Sibling (m or f) |  |  |  |  |  |  |
| Sibling (m or f) |  |  |  |  |  |  |
| Sibling (m or f) |  |  |  |  |  |  |
| Sibling (m or f) |  |  |  |  |  |  |
| Sibling (m or f) |  |  |  |  |  |  |
| Sibling (m or f) |  |  |  |  |  |  |
| Sibling (m or f) |  |  |  |  |  |  |
| Sibling (m or f) |  |  |  |  |  |  |
| Sibling (m or f) |  |  |  |  |  |  |
| Sibling (m or f) |  |  |  |  |  |  |
| Sibling (m or f) |  |  |  |  |  |  |
| Sibling (m or f) |  |  |  |  |  |  |

*Husbands are listed most recent first.

**Information about Husband/ father of child**

| Name of husband/father of child | Date married (or start of relationship) | Date of end of marriage (or end of relationship) | Husband/father of child is father to how many children? |
| --- | --- | --- | --- |
|  |  |  |  |
|  |  |  |  |
|  |  |  |  |
|  |  |  |  |
|  |  |  |  |
|  |  |  |  |
|  |  |  |  |
|  |  |  |  |

**Childhood Health of Interviewee**

| Age | Major Illness | Treatment |
| --- | --- | --- |
|  |  |  |
|  |  |  |
|  |  |  |
|  |  |  |
|  |  |  |
|  |  |  |
|  |  |  |
|  |  |  |
|  |  |  |
|  |  |  |

**Avoiding Pregnancy**

| How do you avoid pregnancy? | How long have you used or did you use this method? | Where do you obtain or learn about these methods of contraception? |
| --- | --- | --- |
|  |  |  |
|  |  |  |
|  |  |  |
|  |  |  |
|  |  |  |
|  |  |  |
|  |  |  |
|  |  |  |
|  |  |  |
|  |  |  |

**Pregnancies**

| # of Pregnancies | Date of delivery | Name of this child | Name of father of child | Duration of pregnancy | Where did you give birth and who assisted? | Current health status of this child |
| --- | --- | --- | --- | --- | --- | --- |
| Pregnancy 1 |  |  |  |  |  |  |
| Pregnancy 2 |  |  |  |  |  |  |
| Pregnancy 3 |  |  |  |  |  |  |
| Pregnancy 4 |  |  |  |  |  |  |
| Pregnancy 5 |  |  |  |  |  |  |
| Pregnancy 6 |  |  |  |  |  |  |
| Pregnancy 7 |  |  |  |  |  |  |
| Pregnancy 8 |  |  |  |  |  |  |
| Pregnancy 9 |  |  |  |  |  |  |
| Pregnancy 10 |  |  |  |  |  |  |
| Pregnancy 11 |  |  |  |  |  |  |
| Pregnancy 12 |  |  |  |  |  |  |
| Pregnancy 13 |  |  |  |  |  |  |
| Pregnancy 14 |  |  |  |  |  |  |
| Pregnancy 15 |  |  |  |  |  |  |

**Questions about becoming pregnant**

| Have you had periods of time when you wanted to be pregnant but were not? |  |
| --- | --- |
| How long did each of these periods last? |  |
| If you were ever concerned that you were not becoming pregnant what did you do to help you become pregnant? |  |
| How many children is a good number for a woman to have? |  |
| What can a girl do to make sure that she is able to have healthy pregnancies and babies when she is a woman? |  |
| What does a woman do to prepare for motherhood before pregnancy? |  |
| What precautions during a pregnancy should a woman take to produce a healthy baby? |  |
| Where are you planning to give birth the next time you are pregnant? |  |
| Who would you like to attend the delivery with you? (sisters, mothers, etc?) |  |

To be completed after interview.

Gravid _____________ Parity ______________ Abortus ____________

Gravid = The number of times a woman has ever been pregnant

Parity = The number of viable births (more than 20 weeks)

Abortus = The number of pregnancies lost for any reason
