# Supplementary material for: Cross-sectional interview study of fertility, pregnancy, and urogenital schistosomiasis in coastal Kenya: Documented treatment in childhood is associated with reduced odds of subfertility among adult women
Source: PLoS Negl Trop Dis. 2017 Nov 27;11(11):e0006101. doi: 10.1371/journal.pntd.0006101 (PMC5720807; doi:10.1371/journal.pntd.0006101)
Supplement: S3 Text — (DOCX) [file pntd.0006101.s004.docx]

**DATA MANAGEMENT AND ACCESS PROTOCOL**

**Roles and responsibilities**

The first author, Sarah Miller-Fellows is responsible for all data management and data storage and be responsible for adherence to the data sharing plan.

**Expected data**
The proposed research produced texts, such as fieldnotes and transcribed interviews and focus groups in English. Metadata for this project includes descriptions of data collection procedures and a codebook for all codes used in the project.

**Period of data retention**

The deidentified transcripts and other data will be shared on a case-by-case basis at the discretion of the first author. All deidentified data will be kept by the researchers in perpetuity. Audio recordings will not be shared outside the research team. Audio recordings will be destroyed within five years of the original study. All data with identifiers will be kept in password-protected encrypted files on the authors work computers.

**Data format and dissemination**

All de-identified text data will be kept in Dedoose, a web-based data management software. This software allows all files to be exported into a standard Microsoft Office format (based on file type) in order to better enable data sharing. During the completion of the first author’s dissertation, deidentified transcripts, fieldnotes and photographs will be shared on a case-by-case basis at her discretion.  Audio recordings will not be shared outside the research team due to confidentiality issues. The study authors will retain all intellectual property rights to the data.

We intend to share data with scientific and non-scientific communities. Upon completion of the field research, relevant findings will be revised and presented to boards at the Msambweni Kenya Hospital and its affiliated health centers.

After publication of this paper, Microsoft Office versions of all deidentified data, in depth descriptions of data collection procedures, and codebooks will be exported from Dedoose and made available in a web archive so that other researchers may access them. Data and metadata will be uploaded the Inter-university Consortium for Political and Social Research (<http://www.icpsr.umich.edu/icpsrweb/landing.jsp>) at the University of Michigan, a social science web archive, where it will be kept in perpetuity. The final version of the first author’s MPH thesis will be made available through Case Western Reserve University's Electronic Theses and Dissertations collection.

.
